# Negative drift of sedation depth in critically ill patients receiving constant minimum alveolar concentration of isoflurane, sevoflurane, or desflurane: a randomized controlled trial

**DOI:** 10.1186/s13054-021-03556-y

**Published:** 2021-04-13

**Authors:** Adrian-Iustin Georgevici, Theodoros Kyprianou, Jennifer Herzog-Niescery, Livia Procopiuc, Sivakkanan Loganathan, Thomas Peter Weber, Martin Bellgardt

**Affiliations:** 1 St. Josef-Hospital, University Hospital of Ruhr-University of Bochum, Bochum, Germany; 2 Medical School, University of Nicosia, Nicosia, Cyprus; 3 Paediatric Intensive Care Unit – Evelina London Children’s Healthcare, Guy’s and St. Thomas, Westminster Bridge Road, London, UK

**Keywords:** Critical care, Desflurane, Electroencephalography, Inhalational sedation, Isoflurane, Narcotrend, Sevoflurane

## Abstract

**Background:**

Intensive care unit (ICU) physicians have extended the minimum alveolar concentration (MAC) to deliver and monitor long-term volatile sedation in critically ill patients. There is limited evidence of MAC’s reliability in controlling sedation depth in this setting. We hypothesized that sedation depth, measured by the electroencephalography (EEG)-derived Narcotrend-Index (burst-suppression N_Index 0—awake N_Index 100), might drift downward over time despite constant MAC values.

**Methods:**

This prospective single-centre randomized clinical study was conducted at a University Hospital Surgical Intensive Care Unit and included consecutive, postoperative ICU patients fulfilling the inclusion criteria. Patients were randomly assigned to receive uninterrupted inhalational sedation with isoflurane, sevoflurane, or desflurane. The end-expiratory concentration of the anaesthetics and the EEG-derived index were measured continuously in time-stamped pairs. Sedation depth was also monitored using Richmond-Agitation-Sedation-Scale (RASS). The paired *t*-test and linear models (bootstrapped or multilevel) have been employed to analyze MAC, N_Index and RASS across the three groups.

**Results:**

Thirty patients were recruited (female/male: 10/20, age 64 ± 11, Simplified Acute Physiology Score II 30 ± 10). In the first 24 h, 21.208 pairs of data points (N_Index and MAC) were recorded. The median MAC of 0.58 ± 0.06 remained stable over the sedation time in all three groups. The *t*-test indicated in the isoflurane and sevoflurane groups a significant drop in RASS and EEG-derived N_Index in the first versus last two sedation hours. We applied a multilevel linear model on the entire longitudinal data, nested per patient, which produced the formula N_Index = 43 − 0.7·h (*R*^2^ = 0.76), showing a strong negative correlation between sedation’s duration and the N_Index. Bootstrapped linear models applied for each sedation group produced: N_Index of 43–0.9, 45–0.8, and 43–0.4·h for isoflurane, sevoflurane, and desflurane, respectively*.* The regression coefficient for desflurane was almost half of those for isoflurane and sevoflurane, indicating a less pronounced time-effect in this group.

**Conclusions:**

Maintaining constant MAC does not guarantee stable sedation depth. Thus, the patients necessitate frequent clinical assessments or, when unfeasible, continuous EEG monitoring. The differences across different volatile anaesthetics regarding their time-dependent negative drift requires further exploration.

*Trial registration*: NCT03860129.

**Supplementary Information:**

The online version contains supplementary material available at 10.1186/s13054-021-03556-y.

## Background

In the past decades, the use of inhalational anaesthetics, such as isoflurane, sevoflurane, or desflurane, has been extended from the operating room to long-term sedation of critically ill patients. It has been documented that volatile sedation offers cardio-protection, minimal metabolism, and shorter emergence times than classic intravenous sedation [1].

Another advantage is seamless monitoring at the bedside: the end-expiratory minimal alveolar concentration (MAC) reflects, at steady-state, the gas concentration in the brain [2]. The MAC at which half of the patients do not respond to verbal commands is known as MAC_awake_. For isoflurane, sevoflurane, or desflurane, the reported MAC_awake_ values are 0.38, 0.34, and 0.34, respectively [2, 3].

Although the recent analgosedation guidelines advocate for minimal sedation and sufficient analgesia to mitigate long-term deep sedation’s detrimental effects, the indications for more profound sedation still vary across hospitals [4]. The task of optimizing sedation depth to simultaneously avoid the complications of excessive sedation and prevent traumatic awareness is a challenging one, especially given the heterogeneity of ICU patients.

Regarding the ICU, most authors chose to deliver variable MAC values around 0.5, above the MAC_awake_ threshold [5–8]. Nonetheless, we found no study related to our primary hypothesis: despite stable age-adjusted MAC, the sedation depth deepens over time. Secondary, we investigate this downdrift trend across the three subgroups (isoflurane, sevoflurane, and desflurane).

## Methods

### Ethics statement

The Institutional Review Board of the Ruhr University Bochum (No. 4780-13, Chair Prof. M. Zenz) approved the study, registered at ClinicalTrials.gov (NCT03860129, on 24 September 2018). This study was conducted in complete compliance with the Declaration of Helsinki (2013), and all patients provided written informed consent before study participation.

### Inclusion and exclusion criteria

Inclusion criteria: adult patients below 80 years of age, non-pregnant, with an American Society of Anaesthesiologists classification I–III, without a language barrier, scheduled for major abdominal, vascular, or orthopaedic interventions, requiring postoperative invasive ventilation due to surgical indications or cardiopulmonary impairment. The exclusion criteria were contraindications to volatile anaesthetics such as brain injuries, increased intracranial pressure, neuromuscular diseases, and malignant hyperthermia.

### Randomization

After ICU admission, the primary investigator randomized patients using the closed-envelope method, ten envelopes for each volatile anaesthetic: isoflurane, sevoflurane (both AbbVie, Ludwigshafen, Germany), or desflurane (Baxter, Unterschleissheim, Germany).

### Sedation delivery, clinical and EEG-based depth assessment

MIRUS™ (TIM, Koblenz, Germany) delivered inhalational sedation by employing an inhalational anaesthetic reflector, which features a controller to maintain the age-adjusted MAC pre-set target, regardless of changes in flow and minute-ventilation [9, 10]. The device continuously recorded the concentration of isoflurane, sevoflurane, or desflurane. Age-adjusted MAC was kept constant at around 0.55. We aimed towards balanced number of observations per patient and subgroup, and since the time under VA is not easily foreseeable, the maximal observation period was limited to 24 h.

Richmond-Agitation-Sedation-Scale (RASS) was documented by nurses every 2–4 h; a physician assessed the first and last RASS values. The analgesia was frequently titrated to achieve a Behavioural Pain Scale (BPS) < 3 in all patients.

The EEG monitor, Narcotrend-Compact M ICU Version 3.2 (Hannover, Germany), logged the sedation depth every minute with a dimensionless number (N_Index) between 0 (flat line) and 100 (full electrical activity, awake) [11, 12].

The N_Index are grouped into the following stages: A awake (95–100 N_Index), B sedated (80–94), C light anaesthesia (65–79), D general anaesthesia (37–64), E0 to E1 general anaesthesia with deep hypnosis (20–36), and E2 to F1 general anaesthesia with increasing burst-suppression (0–19). Stages E and F are associated with increased frequency and duration of burst-suppression and are considered excessive sedation for the ICU patient; stages C and D are considered moderate sedation.

### Sample size estimation and statistical analysis

We used G*Power (Heinrich-Heine-University, Dusseldorf, Germany; version 3.1.9.4) to compute the power sample of a paired *t*-test with α 0.05 and effect size 0.6 on two dependent means: N_Index of the same patients, the mean value in the first and last 2 h of sedation. For a sample size of 30 patients, the test had a power of 0.89. We used the open-source R programming language 3.6.1 for data pre-processing, visualizations, and statistical analyses.

The Kolmogorov–Smirnov test was applied to test the normality of the distribution of all continuous variables. Continuous variables with normal distribution are presented as mean ± standard deviation; non-normal variables are reported as median ± interquartile range.

For the analysis of the MAC, RASS and N_Index values in the first versus last two sedation hours, the one-tailed paired (per patient) *t*-test was applied. The Kruskal–Wallis and Dunn’s tests were used to compare two or three groups of variables that were not normally distributed. The frequencies of categorical variables were compared using *χ*^2^. A value of *p* < 0.05 was considered significant.

Since the sedation duration is not foreseeable, the number of measurements varies across patients and groups. Therefore, we employed two approaches to address this heterogeneity and analyze all longitudinal data: equal bootstrapping per patient and multilevel regressions.

Bootstrapping refers to random sampling with replacement and assigns each patient the same importance in the model [13]. For each of the three VA subgroups, ten thousand bootstrapped linear regressions were performed. To minimize the time-series autocorrelation, ten random samples were drawn per iteration from each patient. The comparison across study arms is achieved with equally sampled bootstrapping per patient and group.

The multilevel linear regression has the clustering per patient as random effect (random intercept and slopes) and sedation duration as fixed effect. Since these models require normal distribution of random (patient) effects, a nested hierarchical structure VA group/patient was not feasible [14]. The Shatterwaite method was used to extract the *p*-values from the hierarchical models. A non-linear relationship between sedation duration and N_Index using generalized additive mixed models is considered if a better *R*^2^ is achieved without overfitting.

## Results

### Number of measurements

After processing the MIRUS™ and Narcotrend logs and excluding incomplete measurements, a total of 21.208 time-stamped pair observations (N_Index and MAC) were obtained. The number of observations per patient was 1019 ± 430, 852 ± 797, and 1158 ± 436 for the isoflurane, sevoflurane, and desflurane groups. The duration of sedation per group in rounded hours was comparable across groups: 18 [16–21]; 17 [10–37]; 18 [15–22] for isoflurane, sevoflurane, and desflurane, respectively (*p* = 0.71).

### Demographics and secondary variables

**Fig. 1:**
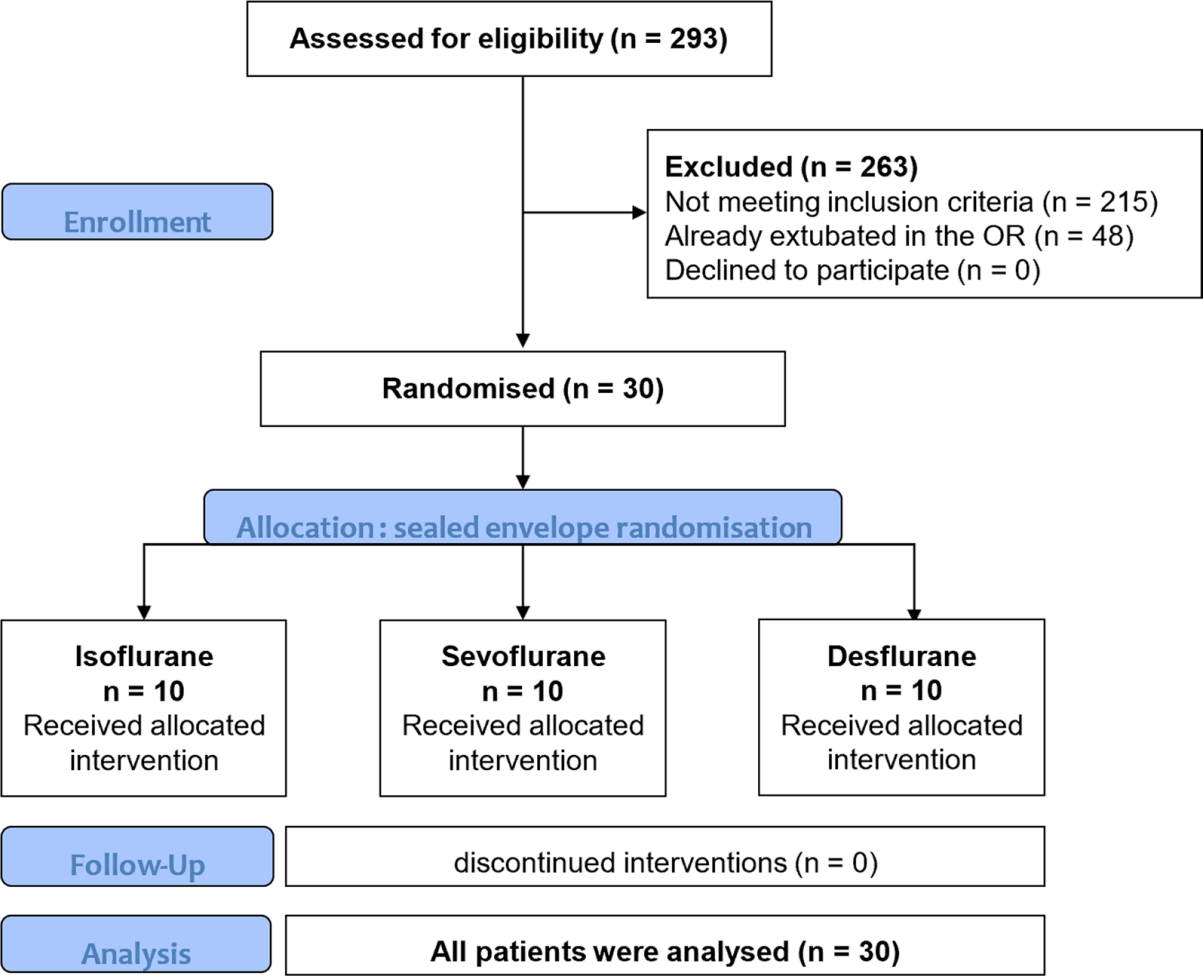
CONSORT diagram for our single-centered randomized controlled trial

Seventy-eight patients underwent major surgeries with an expected longer ICU stay and met the inclusion criteria of eligibility. Of these, 30 patients required postoperative mechanical ventilation. The included patients required postoperative invasive ventilation because they met at least one of the following criteria: surgical indication for strict immobility (after extensive aortic or spinal surgery), increased opioid requirements (> 0.15 µg/kg/h sufentanil), reduced oxygenation (PaO_2_/FiO_2_ < 200), or haemodynamic instability. Figure 1 presents the CONSORT diagram; Table 1 contains patient’s demographics, common diseases, surgeries, clinical scores. Laboratory values are presented in the Additional file 1. The variables do not vary significantly across groups and show no linear correlation with the N_Index. Norepinephrine was the only administered vasopressor, without significant differences across groups, values presented in Table 1.

**Table 1:** Patients demographics along with frequent diseases and surgeries

	Isoflurane	Sevoflurane	Desflurane	*p*-value
Gender (male: female)	8: 2	7: 3	5: 5	0.5
Age (years)	65 ± 10	68 ± 10	60 ± 13	0.23
Weight (kg)	85 ± 10	83 ± 21	69 ± 16	0.06
SAPS	29 ± 10	34 ± 10	26 ± 10	0.23
BPS	3.2 ± 0.17	3.1 ± 0.1	3.2 ± 0.14	0.98
Norepinephrine (µg/kg/min)	0.139 ± 0.004	0.095 ± 0.003	0.164 ± 0.004	0.32
Arterial hypertension (*N*)	4	7	7	0.45
Smoking (*N*)	4	4	2	0.69
Renal insufficiency (*N*)	1	1	0	1
Coronary disease (*N*)	2	3	0	0.32
Surgical interventions (*N*)				
Esophagectomy	5	4	2	0.32
Necrotizing fasciitis	0	1	0	1
Aortic surgery	1	1	3	0.57
Pancreatic surgery	2	0	3	0.32
Peritoneal debulking	1	2	1	1
Spondylodesis	1	2	1	1

[N] represents the number of patients undergoing a type of surgery or having a preexisting condition. SAPS is the abbreviation for Simplified Acute Physiology Score II, and BPS for Behavioral Pain Scale. Kruskal–Wallis was applied on continuous variables and Chi-squared test for nominal data.

### Analgosedation

In the operating room, the patients received epidural analgesia if they had no contraindications. In the isoflurane, sevoflurane, and desflurane groups, the number of patients with epidural ropivacaine was 3, 2, and 3, respectively. General anaesthesia was induced with 0.2 µg/kg sufentanil and 2 mg/kg propofol. For anaesthesia maintenance, patients received sevoflurane MAC 1.12 ± 0.18 and sufentanil 0.17 ± 0.10 µg/kg ideal body weight/hour, without significant differences across groups. Postoperatively, the patients were switched to intravenous sedation with propofol for at least 1 h to allow all accumulated sevoflurane to be exhaled.

In the ICU, we aimed for a BPS ≤ 3 using epidural analgesia (ropivacaine 0.2% + sufentanil 0.75 µg/mL 4 ± 2 mL/hour), anti-inflammatory agents, and intravenous sufentanil µg/kg ideal body weight/hour: 0.25 ± 0.09, 0.26 ± 0.29, and 0.21 ± 0.14 in the isoflurane, sevoflurane, and desflurane groups, respectively (*p* = 0.83). The analgosedation was supplemented before possible stress-inducing medical interventions or nursing care with sufentanil 5–15 µg per bolus, a propofol bolus of 1 mg/kg was administered only five times in all patients during the study period with no statistical difference between groups.

The RASS assessment was performed either before or at least one hour after these boluses.

The measured end-expiratory MAC did not vary significantly: 0.58 ± 0.03, 0.56 ± 0.07, and 0.58 ± 0.06 for isoflurane, sevoflurane, and desflurane, respectively (*p* = 0.32). The electronically logged N_Index was: 33 [28 to 44], 36 [30 to 45], and 37 [31 to 42] for isoflurane, sevoflurane, and desflurane, respectively (*p* = 0.67).

**Fig. 2:**
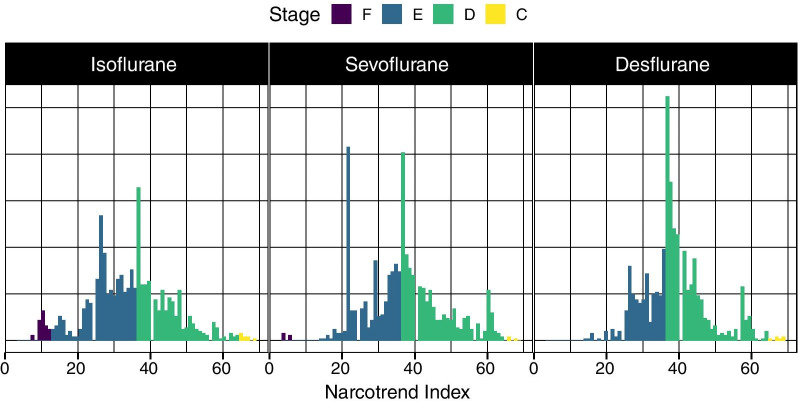
bootstrapped histograms—each patient and group equally represented. On horizontal axis—the N_Index, the vertical axis displays the relative sampling frequency of a certain N_Index value

In the bootstrap histograms, each patient is equally represented; the corresponding plots are in Fig. 2. No patient was in stage A or B, the ratio of moderate versus deep sedation depth was 40%/60%, 52%/48%, and 67%/33% in the isoflurane, sevoflurane, and desflurane groups, and the *χ*^2^ test comparing isoflurane and sevoflurane showed no statistically significant differences (*p* = 0.08). The *χ*^2^ of the sevoflurane—desflurane and isoflurane—desflurane comparisons show *p*-values of 0.03 and 0.0001. Despite comparable MAC values across groups, the deep stages D and F were less frequent in the desflurane group than in the isoflurane or sevoflurane groups.

### Sedation depth assessment in the first and last 2 h

The patient’s MAC values showed no significant difference between the first and the last 2 h of sedation (*p* = 0.55). Across all measurements, the median MAC was 0.58 [0.53 to 0.59].

**Fig. 3:**
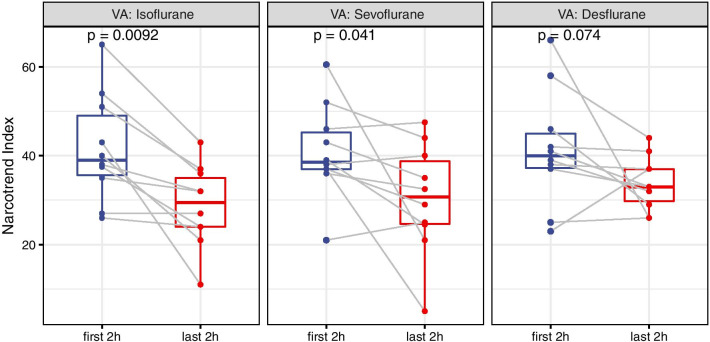
Graphical representation of the pairwise (per patient) *t*-test in the first 2 h (left blue whisker) and last two sedation hours (right red whisker); each patient is representing by a grey line connecting two dots (patient’s median N_Index)

Despite stable MAC values, the mean N_Index in the isoflurane group dropped with a median of − 13.75 [− 4 to − 19] points (*p* < 0.01). In the sevoflurane group, N_Index dropped − 8 points (0.25 to − 13; *p* = 0.04). The difference in the desflurane group -5.55 [− 1 to − 12] was statistically not significant (*p* = 0.07). These results are displayed in Fig. 3.

As presented in Table 2, the RASS values in the isoflurane and sevoflurane group were significantly lower in the last two sedation hours than in the first 2 h; in the desflurane group, the RASS values were comparable per patient.

**Table 2:** RASS values in the first and last two sedation hours

VA group	First 2 h	Last 2 h	*p*-value
Isoflurane	− 3 (− 3; − 3)	− 4 (− 4; − 3)	0.02
Sevoflurane	− 4 (− 4; − 3)	− 4 (− 4; − 3.25)	0.04
Desflurane	− 3 (− 3.75; − 3)	− 3.5 (− 4; − 3)	0.15

The RASS values are expressed as median [1st quartile; 3rd quartile].

The applied statistical test is the pairwise (per patient) *t*-test.

### Analysis of longitudinal data

In the second step of the analysis, we applied an autoregressive hierarchical linear model to all 21.208 longitudinal measurements, with clustering per patient. This regression identified a robust negative correlation between time under sedation and the N_Index (*R*^2^ = 0.76): the regression model is presented in Table 3. Both the linear intercept and slope have a significant *p*-value < 0.001. The regression formula: N_Index = 43 − 0.72 × h, which translates into a N_Index drop of approximately − 17 points after one inhalative sedation day.

**Table 3:** The hierarchical linear model

Terms	Estimates	Confidence-intervals
Intercept	42.85^*^, *p* < 0.001	39.05 to 45.65
Slope	− 0.72^*^, *p* < 0.001	− 1.00 to − 0.44

The hierarchical regression (patient-clustered) is applied to all patients, using 21,208 paired measurements; the *R*^2^ is 0.76

**Fig. 4:**
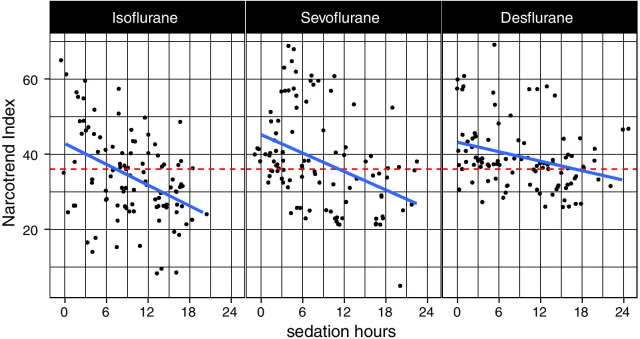
The bootstrapped regression’s mean coefficients per group (slope and intercept) are displayed as blue lines. The horizontal axis is the duration of uninterrupted volatile sedation; the vertical axis is the EEG-derived sedation depth. Below the dotted red line, it is considered excessive sedation (stage E and F)

In the last step of the statistical analysis, bootstrapped linear regressions estimated the effect of time on N_Index for each study group; results presented in Table 4 and Fig. 4. All three model’s intercepts and slopes had *p*-values < 0.01. The slope of desflurane—0.4 × h is almost half of isoflurane (− 0.9 × h) and sevoflurane (− 0.8 × h). The N_Index downdrift in the desflurane group is less pronounced than in patients receiving isoflurane or sevoflurane.

**Table 4:** Statistical output of the bootstrapped linear regressions per subgroup

VA group	Rounded formula	Intercept	Slope	Mean *p*-value	Mean *R*^2^
Isoflurane	43–0.9 × h	42.82 ± 2.28	− 0.92 ± 0.21	< 0.01	0.58
Sevoflurane	43–0.8 × h	45.19 ± 1.96	− 0.82 ± 0.19	< 0.01	0.62
Desflurane	43–0.4 × h	43.18 ± 1.51	− 0.42 ± 0.13	< 0.01	0.71

## Discussion

### Sedation depth drifts downward over time

This study’s primary focus was to examine the difference in the N_Index between the first and last 2 h of sedation. All statistical tests confirmed the sedation time-dependent down-drift in the isoflurane and sevoflurane groups on the EEG-derived index. In the desflurane group, the N_Index drop was smaller than in the isoflurane or sevoflurane study arm. The RASS values analysis grouped per patient and group conveys the same interpretation as the N_Index longitudinal analysis.

In this study, we have chosen MIRUS™ for its ability to automatically maintain stable end-expiratory MAC independent of changes in ventilation parameters, whereas other VA delivery systems like AnaConDa™ can be only manually adjusted. However, given stable MAC, the EEG downward drift cannot be linked to any specific VA delivery device.

N_Index downtrend varied among patients; in three patients from each VA group, the EEG values were relatively stable, probably reflecting the heterogeneity of comorbidities and specific pathophysiology. Patient-clustered hierarchical linear regression, which accounts for interindividual differences, revealed sedation duration as the sole independent predictive variable correlated to the N_Index drift. However, the complexity of brain function remains a challenge for every statistical model. At the bedside, the optimal analgosedation is achievable through individual and frequent titration.

Our results highlight that N_Index slowly drifts into excessive sedation depth (the deep stages E and F) when the titration is not performed (constant MAC). This addresses the high importance of frequent clinical sedation-depth assessments, MAC titration, or (when feasible) sedation pauses to prevent the detrimental effects of excessive sedation, as advocated in the international guidelines on analgosedation-delirium prophylaxis in ICU patients [4].

### MAC and the sedation depth

The literature reports a weak drug–response relationship in both inhalational and intravenous sedative agents, partly attributed to polypharmacy and the high pathophysiological heterogeneity of mechanically ventilated patients [16, 17].

A recent investigation of natural sleep reported minimal EEG-derived values of 41 ± 9.8 [18]. Below this range, the critical ill patient remains in long-term sedation in the unphysiologically deep stages E and F, correlated with significant adverse effects. A study on 26 patients receiving short-time sevoflurane anaesthesia (concentrations between 1.04 and 4.43 vol%) reveals a plateau range in the drug-response (vol% vs. N_Index) curve. This plateau is positioned around an N_Index of 40, which is the inflexion between the maximum δ power and the increase in the burst-suppression ratio [19].

In a study which included ten ICU patients receiving isoflurane with MAC values ranging from 0.2 to 0.8 for 24 h, the reported N_Index values were 38 ± 10 [20]. No study to date compared longitudinally the relation MAC to EEG across the three volatile agents.

Interestingly, the N_Index in almost all our ICU patients also started in this range, which corresponds to stage D (moderate sedation); only after multiple sedation hours, most patients reached excessive sedation (stages E and F).

Our observations highlight that solely maintaining constant MAC is insufficient to prevent ICU patients’ slow drift towards excessive sedation depth. However, the individualized sedation titration is even less reliable with intravenous drugs, which have less predictable half-times in critically ill patients. Better sedation is achievable when the feedback loop assessment-titration is fast and accurate; in this regard, the objective EEG measurements could be proven superior to the subjective RASS assessments.

### Differences across isoflurane, sevoflurane, and desflurane groups

The negative regression slope of desflurane is almost half of those in the other two subgroups, suggesting that the time-dependent negative drift is smaller in patients receiving desflurane (Fig. 4). According to the fitted linear equations, after a day of VA sedation, the N_Index is expected to be 33 (desflurane), 25 (sevoflurane) and 21 (isoflurane). Moreover, only 33% of the N_Index observations were in the deep stages, compared to 48% sevoflurane and 60% isoflurane (Fig. 2).

The current study is probably the first, which looks specifically into EEG dynamic changes during the acute postoperative period in ICU patients receiving VA. Desflurane appears to have a more “robust” profile without significant negative drift. Although we do not have a clear explanation for this, we can highlight that desflurane: (a) is known for its rapid awakening times after long-term sedation [15]; (b) has minimal biodegradation and almost unchanged context-sensitive half-times, which contrast to the significantly prolonged half-times of isoflurane/sevoflurane [21]; (c) the blood/gas and brain/blood solubility coefficients are significantly smaller than those of isoflurane and sevoflurane [22].

Given these facts, it seems possible that the increasingly context-sensitive half-times of isoflurane and sevoflurane may enlarge, during long-time sedation, the difference between the end-expiratory concentration and cortical effect-site of VAs. Stable MAC seems less reliable in the isoflurane and sevoflurane groups to maintain stable sedation depth.

### Limitations of the study

Opioids, like sufentanil, also have a sedative effect. An ICU study with an opioid and non-opioid group may provide more accurate results regarding the drug-response over time of inhalative anaesthetics. In contrast to our study, a multi-centre collaboration could help exclude centre-specific effects.

The MIRUS system provided automatic control of the end-expiratory concentration in a given target, independent of the tidal volume or breathing rate. Nonetheless, drastic changes in ventilation (‘patient fighting the ventilator’) combined with the sudden need to deepen the sedation may require an intravenous agent, such as propofol, at the bedside.

The secondary variables are comparable across the study groups, but undetected interactions between different demographic variables cannot be excluded. Although the desflurane group showed a smaller N_Index drift than the other two groups, the sample size was calculated for the study’s primary goal: sedation deepening despite constant MAC. Therefore, given the interindividual heterogeneity of ICU patients (perioperative pathophysiology and comorbidities), the differences across subgroups can be regarded as hypotheses for more extensive studies, with extended observation periods and sedation pauses. Moreover, larger cohorts could find correlations between common ICU comorbidities and the size of EEG drift.

## Conclusions

In our preliminary study, the time under inhalative sedation shows a suppressive effect on both the clinical assessment (RASS) and the EEG-monitoring (N_Index). When relying only on MAC-monitoring, the ICU patients receiving long-term sedation may drift to an excessive sedation depth (stage E and F). In the desflurane subgroup, we observe a smaller negative drift over time.

## Supplementary Information

**Additional file 1. Fig. S1**: On the vertical axis, N_Index values of N_Index between 0 and 70. The values of age, lactate, base-excess, C-reactive-protein, chloride, leucocytes, sodium, Simplified Acute Physiology Score (SAPS II) and sufentanil are on the horizontal axis. The colours red, green, and blue correspond to measurements from patients receiving isoflurane, sevoflurane, desflurane. There was no significant statistical correlation between the presented values and the three subgroups of the study.

## Data Availability

The data is available in tabular format per request to the authors.

## Abbreviations

ICU: Intensive care unit
MAC: Minimum alveolar concentration
EEG: Electroencephalography
RASS: Richmond-agitation-sedation-scale
